# The Midwives Bill

**Published:** 1917-11-24

**Authors:** 


### NURSING AFFAIRS IN IRELAND.

### The Midwives Bill.

The Midwives Bill is on view: it is excellent, and there will be no complaints. The chief artist in its preparation is Dr. Coey Biggar, Medical Commissioner to the Irish Local Government Board, and none will regard it as invidious to say that he is the first authority in the country on child welfare. Dr. Biggar is the author of a book, published this year under the auspices of the Carnegie United Kingdom Trust, dealing with the physical welfare of mothers and children. The book, volume iv. of the series, is a classic, and a perusal of its pages shows that there is not a single operating force for or against infant welfare that has not been exhaustively and scientifically examined. The author's attitude is modestly and fervently expressed in the foreword. Referring to this substantial volume, which he describes as a Report, Dr. Biggar says: "I beg of its readers to look, not to my poor words, but to the great tragedy that lies behind them, the tragedy of infant sickness and death, to concentrate their minds on this, and determine to do all that lies in their power to rectify the wrongs of the helpless." The spirit that inspired these words has also inspired the Midwives Bill, and its passage through Parliament will complete an important stage on the road to reform.

The Board, according to the Bill, is to consist of four "persons" to be appointed by the Local Government Board, two of whom are to represent county and county borough councils, eight medical practitioners to be appointed by the various medical training-schools, and a certified midwife practising in Ireland, "to be appointed by the Lord President of the Council, when, in his opinion, midwives so qualified are available in numbers sufficient to warrant the appointment." As these conditions already apply, presumably their appointment will be made as soon as the Bill becomes law. It is to come into operation on January 1 next. The Board will be invested with the power of issuing certificates to midwives, regulating their training and examinations, and removing names from the Roll. County and borough councils are to be the local supervising authority, and their duties and powers are duly defined.

An important section of the Bill (No. 11) is that dealing with the reciprocal treatment of midwives on the same lines as under the Scottish Act. "Any woman," runs the text, "who produces evidence satisfactory to the Board that she has been trained as a midwife and certified in any other part of His Majesty's Dominions in which there is for the time being in force any Act or ordinance for the certification and registration of midwives under a public authority, and which admits to its register midwives certified under this Act on reciprocal terms, shall, on payment of the like fee as is payable in ordinary cases, be entitled to be certified under this Act: provided that the standard of training and examination required in such other part of His Majesty's Dominions is in the opinion of the Board equivalent to the standard adopted by the Board." In other words, if a certified midwife from Great Britain or the Colonies wishes to practise in Ireland she can obtain registration under this Act, provided that Irish certified midwives are eligible for registration in whichever country she comes from. This will entail an adjustment of Acts of Parliament regulating the practice of midwifery in different parts of His Majesty's Dominions, and any question arising as to the right of a woman to be certified under this Act is to be determined by the Privy Council. I understand that the English Board are quite sympathetic as regards this section.

